# The influence of insoles with a peroneal pressure point on the electromyographic activity of tibialis anterior and peroneus longus during gait

**DOI:** 10.1186/s13047-016-0162-5

**Published:** 2016-08-22

**Authors:** Oliver Ludwig, Jens Kelm, Michael Fröhlich

**Affiliations:** 1 Sportwissenschaftliches Institut, Universität des Saarlandes, Campus Geb. B 8.1, 66041 Saarbrücken, Germany; 2 Chirurgisch-orthopädisches Versorgungszentrum, Rathausstrasse 2, 66557 Illingen, Germany; 3 FG Sportwissenschaft, Erwin-Schrödinger-Strasse, Gebäude 57, 67663 Kaiserslautern, Germany

**Keywords:** Foot orthoses, Orthotic insoles, Gait, Sensorimotor feedback, Surface electromyography

## Abstract

**Background:**

Peroneus longus acts as a foot evertor and pronator, thus ensuring stability of the talocrural joint by curbing inversion movement of the rearfoot. Increased activation of the peroneus longus muscle in the stance phase could have a stabilising effect on the ankle joint. This study aimed to determine whether the activity of the peroneus longus muscle could be increased by the targeted use of a specially formed lateral pressure element in a customised orthopaedic insole.

**Methods:**

This was a laboratory-based study that utilised a randomised crossover design. Thirty-four healthy participants walked along a walkway in neutral footwear wearing a control insole or a sensorimotor insole with a lateral pressure point adjacent to the tendon of the peroneus longus muscle. The electromyographic muscle activity of the peroneus longus and tibialis anterior muscles was measured using surface electromyography. Contact with the ground was recorded via two pressure sensors under the sole of the shoe. Muscle activity during the stance phase was analysed in the time and amplitude domains and compared statistically with paired *t*-tests for both insole types.

**Results:**

In 27 out of the 34 participants, an additional activity peak of the peroneus longus muscle was observed in the loading response phase with the sensorimotor insole, which reached its maximum at 29.7 % (±4.5 %) of the stance phase. When averaged over all 34 participants, the integrated electromyographic output for the peroneus longus in the mid-stance phase revealed a significant higher activity (*p* < 0.001, post hoc power = 0.98, effect size: Cohen’s d = 0.71) with the sensorimotor insole (18.1 ± 11.3 % MVCs) than with the control insole (11.2 ± 7.7 % MVCs). No significant effects were established for the other gait phases or for the tibialis anterior.

**Conclusions:**

An increase of muscle activity of the peroneus longus muscle was observed during the loading response and mid-stance phase, when orthopedic insoles with a lateral pressure point were worn. We conclude that the pressure point changes afferent information and leads to an increased peroneus longus activation in the time interval in which the pressure point exerted pressure on the peroneal tendon.

## Background

In normal gait, the heel touches down in the initial contact phase with a slight inversion (in the order of 3–4° [1]). In the loading response that follows, the body weight is shifted to the foot, the heel transitions to eversion, and pronation occurs in the midfoot area [2]. In this phase, the peroneus longus acts weakly as a foot evertor and pronator, but does contribute to the stability of the talocrural joint by curbing the inversion movement of the rearfoot [3]. During the stance phase of gait, peroneus longus is most strongly activated in the middle and terminal phases [4]. In contrast, tibialis anterior eccentrically decelerates the lowering of the forefoot area after initial contact (“rearfoot rocker”) and thus guarantees controlled touchdown of the foot [5].

Combined with ligaments and joint capsules, muscular activity ensures the functional stability of the subtalar and talocrural joints [3]. Such stabilisation is initiated and controlled by the proprioceptive input from muscle spindles, tendon receptors, joint receptors and mechanoreceptors [6]. Increased inversion movements of the calcaneus in the landing phase of the foot when walking, running, or after jumping, which are not controlled by such muscle activity, may be the cause of lateral sprains of the subtalar or talocrural joint [7, 8]. Supination injuries, such as lateral ankle ligament sprains, are the most common sports injuries [9] and often lead to long-term instability of the subtalar joint [10]. Most ankle sprains occur when a supinating force is exerted on the foot, and simultaneously, an external rotation force is applied to the leg [8].

This injury mechanism outlined above may be brought forward by multiple factors, such as a mechanical instability of the joints or deficits in joint proprioception [11]. The mechanical and sensory properties of the ligaments and the joint capsules are just as responsible for functional joint stability as the sensorimotor circuits of the stabilising muscle groups [12]. Delayed or insufficient activation of the peroneal musculature in particular has been identified as a possible cause of ankle joint instability, and therefore, is a risk factor for supination injuries [3, 12].

Several approaches have proven to counteract instability of the rearfoot. Mechanically stabilising measures and afferent stimulating interventions exist. Both influence the proprioceptive input and thus change muscular activity, improving the functional stability of a joint [7]. Sports shoes that go above the ankle or have a stable heel cup stabilise the rearfoot mostly in a mechanical way by restricting the inversion movement of the talocrural joint [13]. A similar effect is seen with braces that apply stabilising lateral elements made of relatively rigid materials to safeguard the lateral structures involved with joint stability, such as the lateral ligaments [14, 15]. Taping the rearfoot seems to have both a mechanical effect and an effect on muscle activation via the reinforcement of sensory afferents, although not all studies have been able to confirm mechanical or neuromuscular effects to the same extent [16, 17]. Customised orthopedic insoles have mainly mechanical properties by mechanically supporting and cushioning the foot and by controlling the heel-to-toe movement [18]. Lateral wedges are supposed to take the heel bone to an upright or everted position and thus limit inversion [19]. In recent years, new orthopedic insole designs have appeared on the market, which aim to influence muscle activity in a highly specific way by the application of targeted pressure on tendons in the foot through special insoles with wedged elements [20]. These insole designs are known as neuromuscular afferent stimulating insoles, or more commonly, sensorimotor insoles. They are based on the treatment methods used to help children with cerebral palsy [21, 22] and in patients with spasticity [23]. The origins of this therapeutic approach are dynamic ankle-foot orthoses that are mainly used in paediatric populations with hypertonia in order to attain muscular relaxation [22, 24].

More recent insole concepts apply targeted pressure on tendons to trigger reflexes that lead to muscle contraction [21, 25]. The pressure exerted on the skin by the lateral insole element could stimulate different receptor systems, which can trigger a muscle reaction via afferent corticospinal or propriospinal pathways [26–28]. By actively influencing muscle activation, it may be possible to change the movement pattern, which could provide stabilisation to the ankle joint [28].

There is still no evidence of the effectiveness of these insoles on a neurophysiological level. Therefore, this study aimed to investigate whether the activity of the peroneus longus and tibialis anterior muscle in the stance phase can be influenced directly by specific lateral pressure points in customised orthopaedic insoles.

## Methods

### Participants

Thirty-four participants (16 men and 18 women) took part in the study (Table 1). The participants were healthy, neurologically intact, free from symptoms, and had no known injuries to the ankle joint. Possible joint instabilities were ruled out prior to the study using clinical tests (anterior draw, talar tilt test). The local Ethics Commission approved the trial procedure (reference number 12–1). All participants were informed prior to the study, in accordance with the requirements of the Declaration of Helsinki, on the trial objective and trial procedure and gave their written consent.

**Table 1:** Anthropometric foot shape variables and parameters (*NNHt* Normalised Navicular Height truncated, *AI* Arch Index) of the participant group (*N* = 34)

	Mean SD	Range
Age [a]	35.1 ± 15.0	(18.0–61.3)
Height [cm]	174.8 ± 7.3	(157.0–186.5)
Weight [kg]	72.6 ± 12.3	(53.5–110.5)
NNHt [−]	0.27 ± 0.02	(0.24–0.30)
AI [−]	0.25 ± 0.02	(0.21–0.28)

In response to the call for participants, 54 people expressed their interest. As it is well known that the shape of the foot influences the muscular activity of the lower leg muscles [29], only individuals with normal foot posture were included in the study. For the purposes of this study, normal foot posture was defined by Arch Index (AI) values between 0.21 and 0.28 [30] and normalised navicular height truncated (NNHt) values between 0.23 and 0.30 (normal medial longitudinal foot arch and upright rearfoot position [31]).

**Fig. 1:**
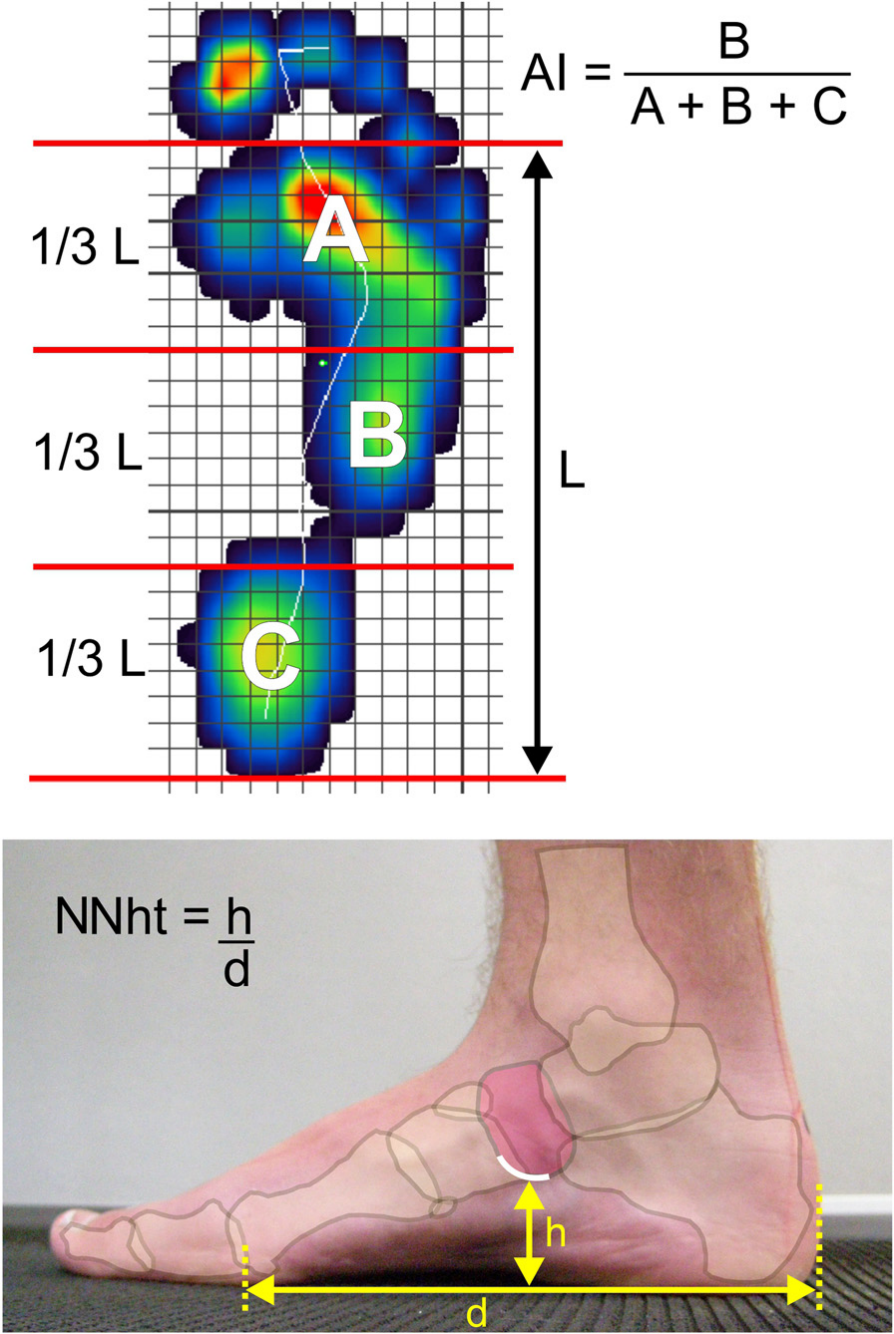
*Top*: foot pressure distribution with reference lines for calculating the arch index. *Bottom*: photograph of the foot with plotted skeleton and reference lines for calculating navicular height truncated. The images are inspired by Murley et al. [29]

Prior to commencement of the study, the Arch Index was determined using a dynamic plantar distribution measurement [32, 33] (PDM platform, Medical GmbH, Isny, Germany) and the normalised navicular height truncated [34] established by photometric assessment of the medial foot arch structure (Fig. 1). Thirty-four interested participants had a neutral foot position and showed no medialised gait line in the pedobarograph, thus they were included in the study.

### Customised insoles

**Fig. 2:**
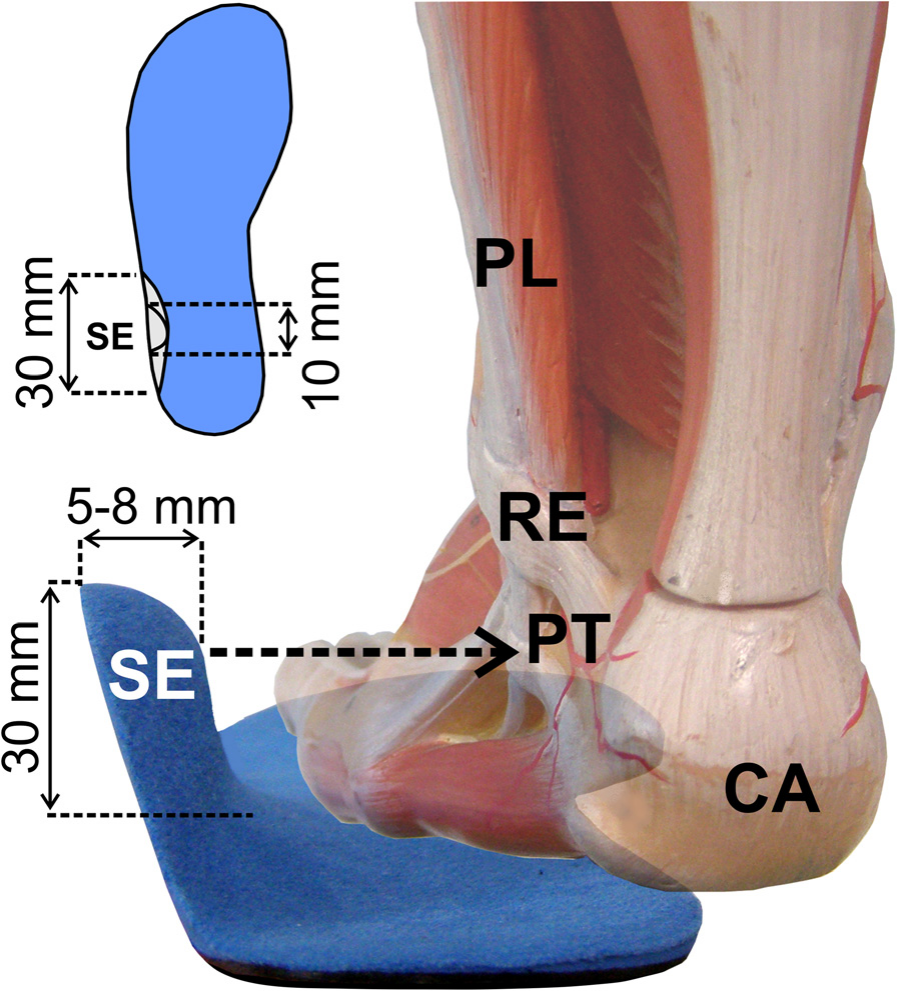
Photomontage of the sensorimotor insole with lateral pressure point (*SE* sensorimotor element) on the tendons (PT) of the peroneus longus (PL) and peroneus brevis muscles; *CA* calcaneus, *RE* retinaculum. The *dotted arrow* indicates the direction of pressure application by the lateral wedge

During an initial appointment, insoles made by Springer Aktiv GmbH in Berlin, Germany were individually adapted to the foot anatomy of each study participant. The insoles were 4 mm thick. A soft foam pressure point (EVA, 35 Shore) was positioned in the lateral rearfoot region as the sensorimotor stimulation point. This pressure point had a concave shape in the plantar area, to prevent mechanical elevation of the outer edge of the foot, and a convex shape in the dorsal area, so that under a load, it was able to exert pressure on the skin overlying the peroneus longus tendon around 8 mm distal to the inferior peroneal retinaculum. The average height of the pressure point was 30 mm, the thickness in the dorsal area 5–8 mm, the length in the plantar area was approximately 30 mm, leveling off dorsally to 10 mm (Fig. 2). The exact measurements varied depending on the foot size. The position of the inferior peroneal retinaculum was palpated below the lateral malleolus, along the lateral aspect of the calcaneus [35], and marked on the skin. To allow for a direct comparison, a control insole was also produced for each participant using the same material, but without the lateral pressure point.

**Fig. 3:**
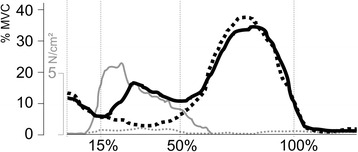
Example of one of the pilot trials showing the average EMG activities of the tibialis anterior muscle with the sensorimotor insole (*solid black line*) and with the control insole (*dashed black line*). The *grey lines* indicate the average pressure values between the lateral wedge and the skin distal to the inferior retinaculum (solid – sensorimotor insole, dashed – control). Values on the x-axis are percentage of the stance phase

The pressure that an insole element exerts on the skin depends on its shape and position and on the shape of the shoe. To ensure that the pressure point used in our study exerted significant pressure, pre-tests were conducted with 12 participants. During their walking, a pressure sensor (GP MobilData, Gebiom, Münster, Germany) measured the pressure curves between the sensorimotor element of the insole (“peroneal pressure point”) and the skin adjacent to the peroneus longus tendon. During the loading response and mid stance phases, pressures ranging between 2.0 and 6.6 N/cm^2^ were recorded, which dropped to 0 N/cm^2^ in the other gait phases. Consequently, a pressure load dependent on the gait phase was confirmed (Fig. 3). Since the pressure sensors themselves exerted a non-definable sensorimotor stimulus due to their bending rigidity and the required wiring, they were not used in the main trials.

In order to exclude the influence of the individual shoe design, all participants wore the same neutral shoe in their correct size. The Adidas Samba basketball shoe was used, which had minimal influence on the position of the rearfoot or midfoot. The shoe has no additional stabilising components in the midfoot and offers excellent forefoot flexibility. To monitor timing and to be able to determine individual gait phases, two pressure sensors were positioned on the sole of the right shoe: (i) at the level of the interphalangeal joint of the hallux and (ii) in the center of the heel (FSR-402, dia. 18 mm, Conrad Electronic).

### Trial sequence

After preparing the skin (shaving, degreasing, roughening), the electromyography (EMG) surface electrodes (Ag/AgCl, Ambu Blue Sensor N) were placed centrally on the bellies of the peroneus longus and the tibialis anterior muscles. Tibialis anterior was also tested as a “control muscle” to be able to provide evidence a possible, targeted effect of the insole element on peroneus longus. Electrode placement and recording technology complied with the SENIAM standard [36]. The EMG signals and the ground contact sensors were recorded by a telemetric EMG system (TeleMyo 2400T, Noraxon, Scottsdale, USA) at 1000 Hz and transferred to a PC after filtration (integrated bandwidth 10–500 Hz).

Prior to starting the trials, one of the investigators randomised 17 participants who would start by wearing the sensorimotor insoles in their shoes, and the other 17 who would start by wearing the control insoles. In order to ensure blinding, the same investigator inserted the insoles in the shoes prior to the test beginning. Neither the participants nor the other investigator who performed the measurements knew whether the functional insole or the control insole was in the shoe. Each trial participant walked with the shoes and insoles at a self-selected speed for a distance of 20 m and did so a total of 6 times (sub-tests 1–3 with insole A, sub-tests 4–6 with insole B). After the third sub-test, the participant walked freely around the room, either barefoot or in socks, for 5 min. While the participant did so, the unblinded investigator switched the insole in the shoe to the second insole to be tested.

The tests were filmed from the rear using a camera positioned close to the ground (Sony HDR-XR-520), so that possible disturbances (stumbling, deceleration) could be identified afterwards.

At the end of the test series, the maximum isometric voluntary contraction (MVC) of the peroneus and tibialis anterior muscles was determined for each participant. For this purpose, whilst sitting and with the foot raised, the participant was asked to perform a maximum isometric pronation movement of the foot (for the peroneus longus) and a maximum isometric dorsiflexion movement of the foot (for the tibialis anterior) against resistance applied by the investigator. The participant was asked to increase the force within 3 s to a maximum and to hold this for 2 s. Three MVC efforts were performed, separated by a recovery period of 1 min. Once the data were collected, 500 ms intervals from the middle of the three MVC recordings were averaged using the root mean square (RMS) [29].

### Evaluation

Sub-tests 1 and 4 were used to allow the participants to become accustomed to the shoes and the insoles. Accordingly, sub-tests 2 and 3, and sub-tests 5 and 6 were the tests that were evaluated. If the gait speed in both sub-tests deviated by more than 5 %, the 3rd or 6th sub-test was evaluated; this was necessary in 4 cases. The EMG data was analysed using *Myo Research Protocol* software (Noraxon, Scottsdale, USA). After rectification and smoothing, the EMG signals were amplitude normalised to the averaged MVC value. The time axis was standardised to the duration of the supporting phase. Then the data from 10 steps taken from the middle of the registered sequences were averaged for the “insole” and “control” variants, respectively, and the 95 % confidence interval calculated.

For each of the two test sequences (“insole” or “control” variant) for each of the participants, the integrated EMG (iEMG) was calculated using the rectified raw data for the time intervals 0–15 % (loading response), 15–50 % (mid-stance) und 50–100 % (terminal stance / push off) for both muscles. This classification was established as in a previous study, the first deviations in muscle activity could be observed at 17 % of the stance phase [25].

The amplitude peak and time of the first (start of stance phase) and second (push-off phase) activation peak of the peroneus longus muscle were determined [3, 29, 37]. If an additional activation peak occurred during the loading response phase, the time and amplitude of this peak were also determined. A statistically significant deviation of the EMG activity was determined as the point at which the 95 % confidence intervals of the averaged values between the “insole” and “control” variants no longer overlapped [29].

### Data analysis

Statistical analyses were performed using the program WinStat (V 2009.1) for Microsoft Excel and the Shapiro-Wilk Test Calculator (SciStatCalc). After checking the normal distribution of the values using the Shapiro-Wilk test and the variance homogeneity using the Bartlett test, the paired *t*-test for dependent samples was used to compare the iEMG values. To rule out confounding effects due to the participants becoming accustomed to the trial conditions, a paired *t*-test was used to calculate the intra-individual differences for the sub-groups “first control, then insole” and “first insole, then control” [38, 39]. An error probability of less than 5 % (*p* < 0.05) was deemed to be statistically significant. Since our study was a ‘proof of concept study’ that was designed to identify whether any effects occur at all, no information on the effect size was known beforehand. Therefore, effect size and power were calculated post hoc using G*Power 3.1.

## Results

The activation pattern for peroneus longus showed peaks at initial contact and at push-off. There were no significant differences in the time or the maximum amplitude of both these activation peaks in the ‘insole’ and ‘control’ test variants (Table 2). At no point during the gait cycle were there any significant differences in the activity of the tibialis anterior muscle.

**Table 2:** Activity of the peroneus longus and the tibialis anterior muscle (mean ± SD), *N* = 34, CI = 95 % confidence intervals for the paired differences

		Control	Sensorimotor insole	Paired difference	95 % CI	*P*-value (2-tailed)
Peroneus longus						
Initial contact	time (% stance phase)	0.48 ± 4.53	1.53 ± 4.54	1.95 ± 2.67	1.05–2.85	0.179
	amplitude (% MVC)	28.78 ± 10.73	27.17 ± 10.09	−1.61 ± 3.43	−2.76– −0.46	0.117
Mid-stance	time (% stance phase)	–	29.67 ± 4.51	–	–	–
	amplitude (% MVC)	16.47 ± 7.51	21.79 ± 9.98	5.33 ± 6.08	3.29–7.37	<0.001
Push-off	time (% stance phase)	74.32 ± 6.04	74.50 ± 8.03	0.18 ± 6.10	−1.87–2.23	0.890
	amplitude (% MVC)	68.96 ± 21.00	65.80 ± 20.66	0.17 ± 7.67	−2.41–2.75	0.920
Tibialis anterior						
Initial contact	amplitude (% MVC) at 0 % stance phase	39.08 ± 9.87	37.04 ± 9.55	2.04 ± 8.27	−0.74–4. 82	0.160
Mid-stance	amplitude (% MVC) at 30 % stance phase	4.40 ± 3.38	4.56 ± 3.31	−0.16 ± 3.63	−1.38–1.06	0.797

**Fig. 4:**
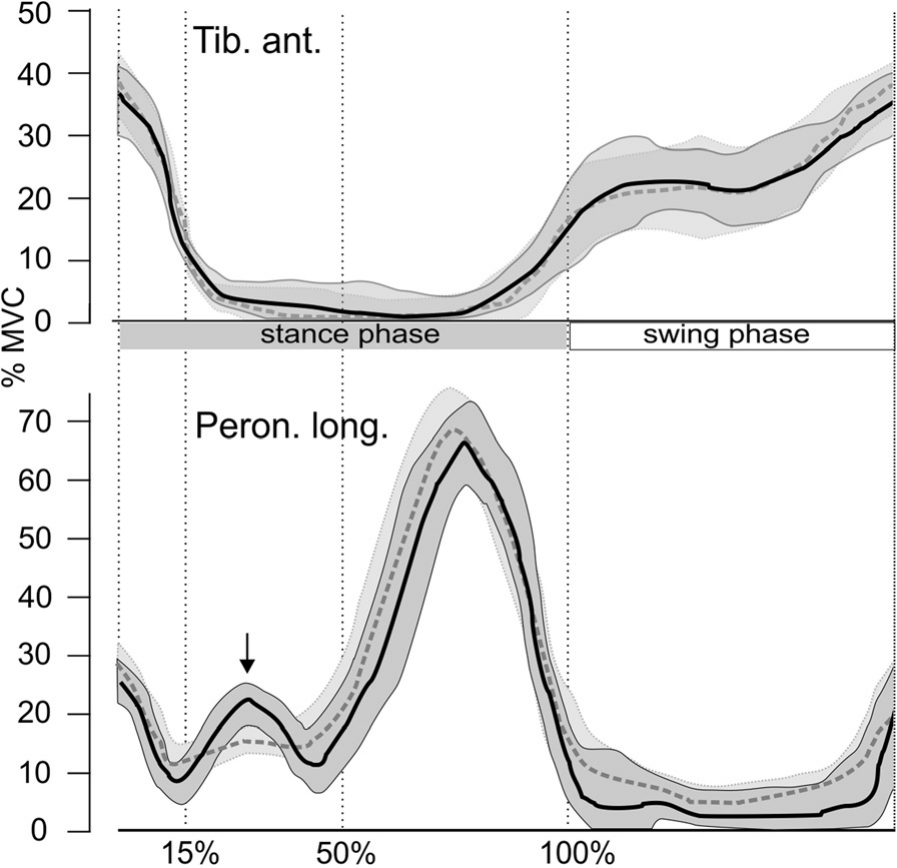
EMG and 95 % confidence intervals (*shaded*) averaged over all 34 participants for the tibialis anterior muscle (*upper*) and peroneus longus muscle (*lower*) of a participant. *Dashed lines* represent the control insole and *solid lines* represent the insole with sensorimotor element. *Vertical lines* indicate the evaluated interval as a percentage of the stance phase duration. The *arrow* indicates the additional activation peak

**Fig. 5:**
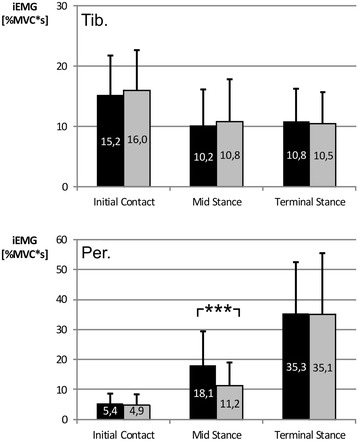
Integrated EMG (mean ± SD) of the tibialis anterior muscle (*upper*) and the peroneus longus muscle (*lower*) for all participants (*N* = 34) in the gait phases. *Black bars* represent the insole with sensorimotor element and *grey bars* represent the control insole. *** *p* < 0.001

In 27 of the 34 participants, an additional activation peak of the peroneus longus muscle occurred in the loading response phase with the sensorimotor insole, which, on average, reached its maximum at 29.7 % (±4.5 %) during mid-stance (Fig. 4). Based on the average of all 34 participants, a significant difference was identified between the EMG amplitudes of the ‘insole’ and ‘control’ variants during this gait phase (Table 2). For peroneus longus in the mid-stance phase, the iEMG revealed a significantly higher activity (*p* < 0.001, post hoc power = 0.98) with the sensorimotor insoles (18.1 ± 11.3 % MVCs) than with the control insoles (11.2 ± 7.7 % MVCs, Fig. 5). The effect size (Cohen’s d) was 0.71, which indicates a large effect. No significant effects were found for the other gait phases or for the tibialis anterior.

No statistically significant differences could be established (*t* = 1.1, *df* = 32, *p* = 0.205) between the intra-individual differences for maximum amplitude in the sub-groups that started the tests with the control insole or the sensorimotor insole; homogeneous variances were present (*F* = 1.842, *df* = 1, *p* = 0.349). Therefore, confounding effects resulting from the order in which insoles were worn could be ruled out [38, 39].

## Discussion

The purpose of this study was to investigate whether specific changes in muscle activity during gait could be generated by so-called sensorimotor insoles. Since we wanted to examine possible basic physiological effects, we chose healthy participants with normal foot posture and function.

In the majority of participants investigated, an increase in the activity of the peroneus longus could be observed in the mid-stance phase when the sensorimotor insoles were worn. The additional activation peak was localised in the first third of the stance phase (*loading response*). However, the activation of the peroneus longus muscle at the start of the stance phase (*initial contact*) and in the push-off phase revealed no differences. Thus we can conclude that the observed effect can be traced to the lateral pressure point in the insole, which exerted pressure on the skin overlying the muscle’s tendon during the loading response phase. In the loading response/mid-stance phase, during which the increased activity of the peroneus longus was observed when sensorimotor insoles were worn, only weak activity of this muscle is generally measurable [2, 3, 37]. Therefore, the effect of the sensorimotor insoles appears to be specific, in the sense that the provoked muscle reaction has a unique time response. Non-specific effects of insoles on the mechanical sensitivity of the foot and the distribution of plantar pressure have already been documented [40, 41, 44].

According to the postulated mode of action of sensorimotor insoles, the insoles function by targeted afferent stimulation and, due to the profile of the lateral pressure points, should not be able to change the position of the foot in a mechanical way. The special concave shape of the lateral pressure point is designed to prevent a purely mechanical modification of the midfoot position in the same way that an outer-edge elevation [21] or a lateral wedge [42]. In this case, the foot would have been moved into an increased pronated position and a reduction of the activity of the foot pronator muscles would have been expected [2]. An activity-boosting action by mechanical medial elevation, as discovered by Murley and colleagues [43], can be excluded by the insole shape because the insoles used in this study did not have any medial supporting elements.

No changes were found in activity of the tibialis anterior muscle, which served as a ‘control muscle’ to exclude any non-specific effects on general muscle activity induced by the insoles. Nevertheless, recordings of the muscle activity by the strongest supinator muscle, the tibialis posterior, would have been more meaningful, however these measurements would only be possible using invasive methods [29]. The observed change in the activity of peroneus longus also needs to be distinguished from the mode of action of adhesive sports tapes or supportive orthoses that are also used to stabilise the ankle. These interventions employ mechanoreceptor stimulation to modify the latency of peroneal activation [45]. They can increase the activation amplitude, but they do not generally generate additional activation patterns [46].

The peroneus longus muscle clearly has a multifaceted function at the start and middle of the stance phase. Thus, Louwerens and co-workers [37] were able to find not only a major variability in activation of peroneus longus at the start of the stance phase (at various points during the walking movement), they were also able to pinpoint additional activation peaks, which they interpreted as the contribution by the muscle to dynamic balance control. Similar results have been reported for patients following an ankle sprain [3]. This emphasises the important role of the peroneus longus for ankle joint stability. Murley and colleagues [29], who compared the muscular activity of lower limb muscles in participants with normal arched feet and flat-arched feet, also recorded increased peroneus longus activity at the beginning (at about 10 %) of the stance phase. The activity peaks found in this study occur later (at about 30 %) and therefore, can be interpreted as a different neuromuscular effect.

With respect to the finding of the activation peaks discovered in the mid-stance phase, in principle, a number of neurophysiological control mechanisms can be considered. For example, corticospinal or propriospinal control circuits [26, 27], which are triggered by modified afferent input. In fact, a number of different receptor systems may be involved here. The pressure exerted on the skin by the lateral insole pressure point can stimulate mechanoreceptors in the dermis and epidermis and nociceptors, which can trigger a muscle reaction via afferent pathways [28]. However, none of the participants reported feeling any pain or unpleasant pressure on the outside of the ankle whilst wearing the sensorimotor insoles, which would be indicative of a nociceptive evasive response. Nevertheless, a minor position change of the calcaneus, which could be caused by the lateral pressure point, could also trigger stimulus responses via joint receptors; for example, in the subtalar joint or the calcaneo-cuboid joint. As the pressure point was positioned over the course of the tendon of the peroneus longus and peroneus brevis muscles, the observed muscular reaction can also be interpreted as the result of a propriospinal tendon reflex [49–52]. In the mid part of the gait cycle, the lateral insole pressure point exerts a measurable pressure along the dorsomedial area of the tendons of the peroneus brevis and peroneus longus, which could lead to stimulation of the muscle spindle receptors. The precise identification of which afferent structures are responsible for the observed reaction cannot be ascertained by the current study and would require further investigations using neurophysiological techniques.

It is not clear whether supination injuries of the ankle can be prevented solely by increased activation of the evertors of the ankle joint, even if delayed peroneus activation has been identified as the cause of ankle sprains [10, 53]. Joint injuries during sports are often the consequence of movements that happen so fast that a reflex response to stabilise the joint comes too late [3] and it is presumed that the primarily intrinsic effects of joint stiffness play a role in injury prevention [54–56]. However, Murley and colleagues [29] were able to show that an anticipatory pre-activation of peroneus longus can be observed especially during rhythmic movements. We were able to confirm this in our study, as an activation peak shortly before and at the beginning of the stance phase (*terminal swing* and *initial contact*) was observed in practically all participants. This can be interpreted from the perspective of a stable foot position before landing [57, 58]. Likewise, a ‘readiness position’ by the musculature can also be considered, as a pre-activated sensorimotor system is able to react faster to disruptive stimuli in this critical gait phase [5, 54]. In this context, additional peroneus longus activation by the insole could have a positive effect on stabilisation of the rearfoot when walking. This might be particularly useful if the sensorimotor control of joint stability is reduced as a result of injury [6, 54]. Further research in this area is recommended.

In the evaluation as to whether the additional activation achieved by the sensorimotor insole has kinematic relevance, comparisons can be drawn with stimulus–response trials. The additional activation peak that occurred in the mid-stance phase reached averaged maximum values of 22 % MVC. Even if this seems low in relation to the muscle activity measurable at push-off, it must be taken into account that functional reactions of the peroneal muscles after landing on a supinating platform lie within a range of 5–20 % [47], and that marked increases in joint inversion are already associated with an elevation in peroneal activity of around 50 % of the activity at rest [48].

At the time of the additional activation peak, at 29 % of the stance phase, bodyweight is transferred to the foot (*loading response*). The question is raised as to whether an effective correction of the rearfoot position will still be possible at this time. However, Delahunt and co-workers [48] were able to measure a functionally effective increase in the activity of peroneus longus during the loading response phase, which occurred in individuals with functional ankle joint instability at increased inversion angles. They interpreted this as a protective mechanism during a critical phase when bodyweight is transferred increasingly via the talocrural joint into the foot. During the phase of the observed additional amplitude maximum, the foot is offered a low level of protection only by the muscle activity, as it is stabilised mechanically across the frontal plane during an axial load [59]. Louwerens and co-workers [37] point out that with external laterally-acting disruptive stimuli, or even with anticipatory compensatory movements as a result of a trunk movement, the peroneal musculature must intervene to stabilise the limb. Thus the elevated peroneus longus activity observed in this study could be helpful for stabilisation of the ankle joint at the time it occurs. This should be the topic of further research.

There are several limitations of this study that also need to be considered. Firstly, the study aimed to investigate the basic effects of sensorimotor insoles on muscle activity. It is not possible to draw conclusions about any other types of intervention for patients with foot deformities or joint instability. Further studies could clarify whether the observed effects occur in the same way in participants with previous ankle sprains, with modified peroneal recruitment [60], or in participants with foot deformities like *pes planus* or *pes cavus*. In particular, athletes with *pes cavus*, who tend toward increased supination [61], could potentially benefit from an evaluation of the effect of the tested insole as a preventive measure, as it is known that increased foot supination is a major risk factor for overuse injuries [62]. Secondly, we only investigated healthy participants, but the underlying physiological mechanisms of peroneal activation might be interesting for the treatment of patients. Thirdly, the results are limited to electromyographic effects of only two muscles of the lower extremity, so we are unable to make broader conclusions about other lower limb muscles. Fourthly, only short-term effects were examined; possible long-term effects of neuromuscular adjustments during prolonged wear of the sensorimotor insoles were not included in the study. Finally, we did not study kinematics, so conclusions about changes in lower limb position and movement cannot be made from our study.

## Conclusion

In this study, we were able to demonstrate that a gait phase dependent increase in the activity of the peroneus longus muscle is possible using a customised orthopaedic insole with a lateral pressure point. Changes of afferent sensory information, which are caused by the pressure from the orthopedic insole, could be responsible for the observed changes in peroneus longus activation. This assumption is based on the fact that activity only increased during the loading response/mid-stance phase and that tibialis posterior activity was not influenced.
